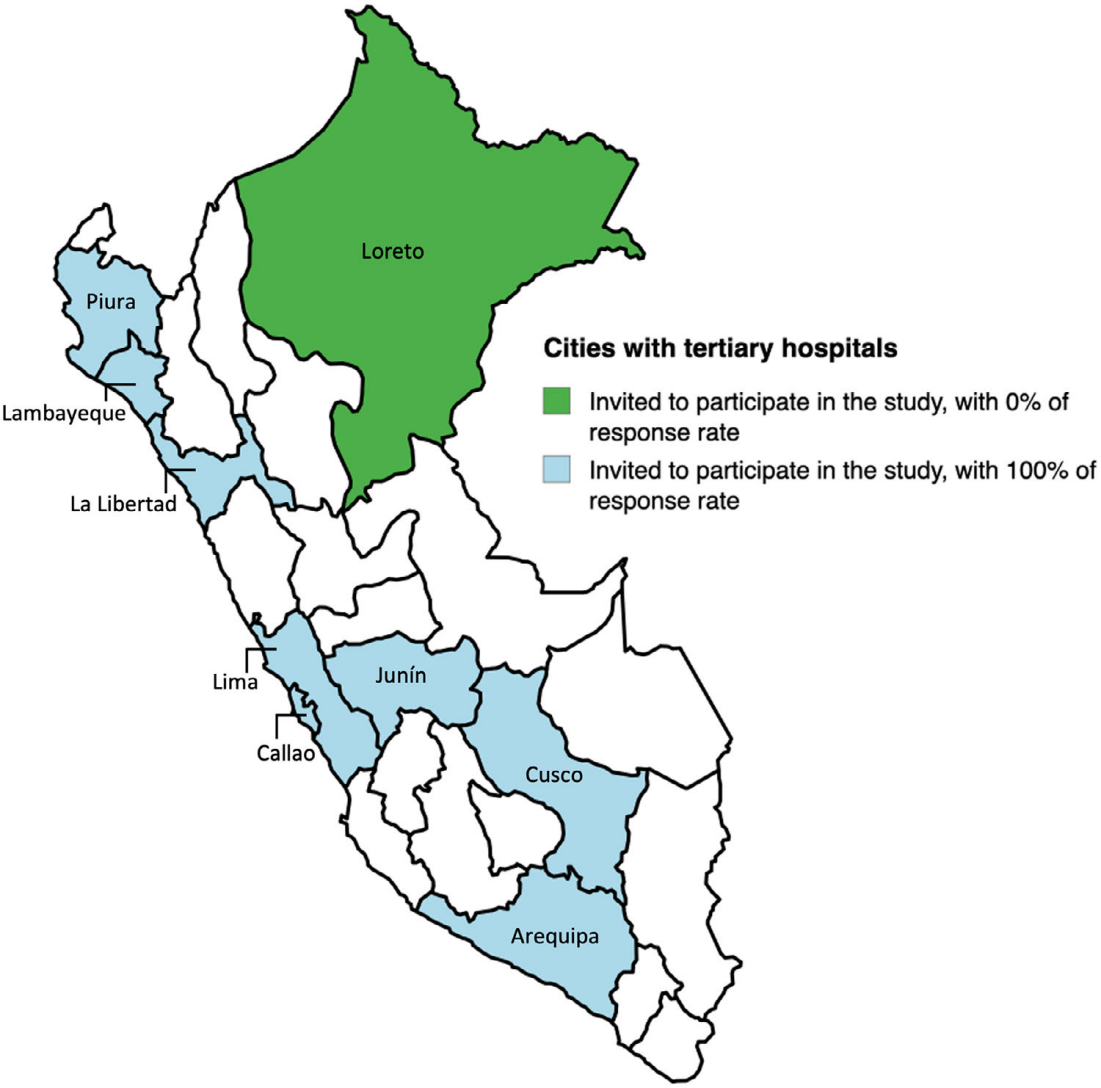# Neurologists’ perceptions about the readiness for implementing anti‐amyloid drugs for Alzheimer's disease in Peru

**DOI:** 10.1002/alz70860_105685

**Published:** 2025-12-23

**Authors:** Belen Custodio, Rosa Montesinos, José Carlos Huilca, Katherine Agüero‐Flores, Diego Bustamante‐Paytan, Diego Chambergo‐Michilot, Graciet Verastegui, Marco Malaga, Mayra Rojas, Isabel Camargo, William Bayona, Nilton Custodio

**Affiliations:** ^1^ Unidad de Investigación de Deterioro Cognitivo y Prevención de Demencia, Instituto Peruano de Neurociencias, Lima, Lima, Peru; ^2^ Unidad de Investigación y Docencia, Equilibria, Lima, Peru., Lima, Lima, Peru; ^3^ Unidad de Investigación de Deterioro Cognitivo y Prevención de Demencia, Instituto Peruano de Neurociencias, Lima, Peru, Lima, Lima, Peru; ^4^ Universidad Científica del Sur, Lima, Perú, Lima, Lima, Peru; ^5^ Hospital Regional Docente, Trujillo, Peru, Trujillo, Peru; ^6^ Hospital Regional Honorio Delgado Espinoza, Arequipa, Peru., Arequipa, Peru; ^7^ Hospital Adolfo Guevara Velasco, Cusco, Peru., Cusco, Peru

## Abstract

**Background:**

Alzheimer's disease (AD) is a neurodegenerative disease affecting millions of people worldwide. This situation is particularly severe in low‐ and middle‐income countries, where numerous barriers hinder timely diagnosis and treatment. To date, two monoclonal anti‐amyloid have shown positive results in phase III clinical trials. However, these medications involve significant complexity in their administration, requiring specialised infrastructure, highly trained professionals, and periodic follow‐ups; which poses a huge challenge for healthcare systems. We aim to explore the perceptions of neurologists in Peru regarding the changes needed to implement the use of monoclonal antibodies in alignment with the clinical practice guidelines.

**Method:**

This cross‐sectional study was conducted in Peru using the key informant (KI) methodology. The KI were neurologists from multiple regions across the country. A comprehensive list of tertiary‐level hospitals (public healthcare system, social security, and police and armed forces) was compiled, and at least one neurologist from each institution was contacted. The instrument used was adapted from a study conducted in Spain, which included questions regarding the changes required in response to the introduction of these medications. It addresses modifications in the diagnostic process and patient care, changes in diagnostic and therapeutic techniques, the impact on public awareness and families following the introduction of these drugs, the resources needed for neurology services, and potential shifts in dementia research. Statistical analysis was performed using Stata18, employing descriptive statistics and frequency distributions.

**Result:**

Figure 1 displays the cities with tertiary‐level hospitals that were invited to participate. Twenty‐eight key informants completed the questionnaire. A consensus was reached regarding the significant impact that the introduction of monoclonal antibodies would have on the functioning of neurology services. Over 85% agreed that neurology services would require an increased number of neurologists and nurses. Additionally, 93% suggested that the implementation of brief diagnostic scales in primary care could be effective and that the frequency of follow‐up visits should increase.

**Conclusion:**

For the introduction of monoclonal antibodies for AD to be feasible in Peru, modifications to the functioning of healthcare institutions are required, emphasizing the need for strategic healthcare planning.